# Acridine yellow G (AYG) as a photo-induced electron transfer (PET) photocatalyst employed for the radical Michael–Mannich cyclocondensation of imines

**DOI:** 10.3389/fchem.2022.1015330

**Published:** 2022-10-06

**Authors:** Farzaneh Mohamadpour

**Affiliations:** School of Engineering, Apadana Institute of Higher Education, Shiraz, Iran

**Keywords:** acridine yellow G (AYG), photo-induced electron transfer (PET), renewable energy source, polyfunctionalized dihydro-2-oxypyrroles, photochemical synthesis

## Abstract

A four-component domino Michael–Mannich cyclocondensation of amines, dialkyl acetylenedicarboxylaes, and formaldehyde was utilized to develop a green technique for sans metal combination of polyfunctionalized dihydro-2-oxypyrroles. It involves visible light as an environmentally friendly power source and acridine yellow G (AYG) as a photo-induced electron transfer (PET) photocatalyst. The motivation behind this examination was to expand the utilization of a non-metal dye that is both reasonable and broadly accessible. Photochemically catalyzed AYG flaunts exceptional returns, energy effectiveness, and natural agreeableness, as well as extraordinary iota economy, efficient highlights, and comfort of purpose. Key abilities consist of an easy experimental setup, big substrate tolerance, finance-friendly, clean painting-up strategies within the absence of tedious separation techniques, and minimized the quantity of waste for each organic transformation. The type of yields is pretty uniform (85–97%, average 92.09%), and the shape of reaction times might be very speedy (15–30 min, average 21.59 min), and the factor stated inside the dialogue is that the method tolerates quite a number electron-donating and electron-withdrawing functional groups, while, however, giving extremely good yields. The response within the reason is insensitive to the person of the substituents. Subsequently, many compounds and natural factors can be followed over the course of time. Shockingly, gram-scale cyclization is conceivable, proposing that the strategy could be utilized in industry.

## Introduction

**FIGURE 1:**
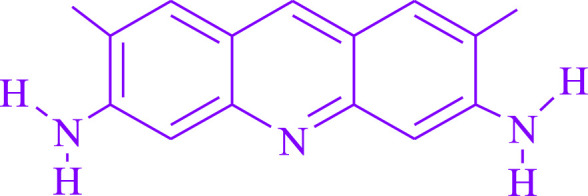
Acridine yellow G structure.

Because of retaining light in the noticeable scope of the electromagnetic range, photo-redox catalyst fosters their stable photoexcited states (Patel et al., 2021). Various flow reactors (Politano and Oksdath-Mansilla, 2018) including atom inexpensive, green, and efficient processes, have been constructed using visible light and dual photosensitized electrochemical methods (Verschueren and de Borggraeve, 2019), (Patel et al., 2021). Acridine yellow G (Figure 1) is a highly fluorescent dye that is widely used in cytology to stain cells (Tangelder et al., 1995; Teuber et al., 2001), DNA staining in chromatography (Vincent and Goldstein, 1981), fluorescent markers (Goryacheva et al., 2000), spectrophotometry to identify residues of impurities (Pérez-Ruiz et al., 1997; Pérez-Ruíz et al., 2003), and photocatalysis (Takemura, 1962; Amat et al., 2007; Arques et al., 2009). AYG has been shown to have photosensitizing (Saint-Cricq et al., 2012) and photodynamic (antibacterial) (Webb et al., 1979) effects (Kostjukova et al., 2021).

As a result of its enormous energy holds, modest expense, and sustainable power sources, green physicists believe noticeable light illumination to be a dependable innovation for harmless to the ecosystem compound combination (Mohamadpour, 2020a; Mohamadpour, 2021a; Mohamadpour, 2021b).

The designs that make up pyrrole subordinates have aroused the curiosity of chemists because of their organic and pharmacological impacts (Lampe et al., 1993; Shiraki et al., 1995; Singh et al., 1995; Borthwick et al., 2002; Chen et al., 2005; Alp et al., 2010). There are numerous options to synthesize polyfunctionalized dihydro-2-oxypyrroles, including I_2_ (Khan et al., 2012), glycine (Mohamadpour, 2020b), AcOH (Zhu et al., 2009), Cu(OAc)_2_.H_2_O (Lv et al., 2013), Fe_3_O_4_@nano-cellulose–OPO_3_H (Salehi and Mirjalili, 2017), tartaric acid (Mohamadpour et al., 2017), nano-Fe_3_O_4_@SiO_2_/SnCl_4_ (Mirjalili et al., 2019), glutamic acid (Mohamadpour, 2019a), graphene oxide (Bavadi and Niknam, 2018), caffeine (Mohamadpour, 2019b), 2,6-pyridinedicarboxylic acid (Khan et al., 2016), saccharin (Mohamadpour et al., 2016), BiFeO_3_ nanoparticles (Singh and Rajput, 2018), and CoFe_2_O_4_@SiO_2_@IRMOF-3 (Zhang et al., 2017). In order to manufacture heterocyclic compounds, we investigated photocatalysts (Mohamadpour, 2021c; Mohamadpour, 2021d; Mohamadpour, 2021e; Mohamadpour, 2022) in the green medium. This concentrate likewise tells the best way to utilize a photo-redox catalyst that is modest and broadly accessible. The photochemical mechanism by which AYG works as a photo-induced electron transfer (PET) photocatalyst has already been reported (Sengupta et al., 2018). Visible light facilitates the Michael–Mannich cyclocondensation of amines, dialkyl acetylenedicarboxylaes, and formaldehyde in ethanol at rt. This reaction was carried out at high speed and yielded.

## Experimental

### General procedure

A solution of amine 1 (1.0 mmol) and dialkyl acetylenedicarboxylate 2 (1.0 mmol) in EtOH (3 ml) was agitated for 15 min in the presence of AYG (1.5 mol%) under blue LED (12 W) irradiation at rt. The reaction mixture was then stirred at rt while the amine 3 (1.0 mmol) and formaldehyde 4 (1.5 mmol) were added. TLC was used to track the response. Thin layer chromatography (TLC) was carried out with silica gel as the stationary phase utilizing EtOAc/*n*-hexane (1:2) as an eluent. After the reaction, the resulting product was screened and washed with ethanol to produce the pure chemical without additional purification. Regardless of whether we could blend the previously mentioned synthetic substances utilizing gram-scale advancements, we needed to check whether we could increase to the level expected for drug process R&D. In one examination, 50 mmol aniline, 37.5 mmol formaldehyde, and 25 mmol diethyl acetylenedicarboxylate (DEAD) were used. The enormous scope response proceeded according to the plan, taking simply 20 min to complete, and then the item was accumulated utilizing standard filtration techniques. This substance’s ^1^HNMR range demonstrates that it is spectroscopically unadulterated. The items were ordered subsequently looking at spectroscopic information (^1^HNMR). For this composition, the spectroscopic information is given in the Supporting Information file.

## Results and discussion

**SCHEME 1:**
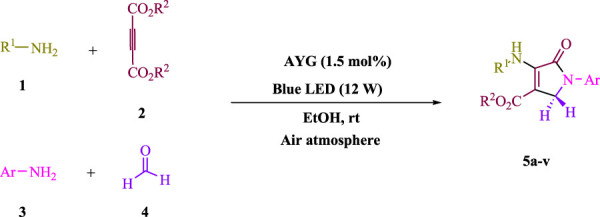
Synthesis of polyfunctionalized dihydro-2-oxypyrroles.

To start, the condensation of formaldehyde, aniline, and dimethyl acetylenedicarboxylate (DMAD) is investigated. The reaction was carried out at room temperature, in EtOH (3 ml), and utilizing LED light. In the absence of a photocatalyst, a trace amount of product was produced. Acridine yellow G, xanthene, riboflavin, 9*H*-xanthen-9-one, rhodamine B, alizarin, phenanthrenequinone, acenaphthenequinone, rose bengal, erythrosin B, and fluorescein were all attempted in the identical settings to improve the response. With yields ranging from 42 to 97%, this reaction generated the approved matching product **5a**. As per the discoveries, AYG performed better in such a response. The yield was expanded to 97% utilizing 1.5 mol% AYG (Table 1, entry 3). H_2_O, DMF, DMSO, THF, DCM, toluene, and solvent-free conditions generally brought about lower item yields, as displayed in Table 2. In MeOH, EtOAc, and CH_3_CN, the yield and reaction rate improve. The reaction occurred in EtOH with a high rate and yield. Under indistinguishable circumstances, a yield of 97% was delivered, as displayed in Table 2. A variety of light sources were used to investigate how blue light influences yield. There was a minuscule measure of **5a** without utilizing the light source, as per the test control. As per the discoveries, light and AYG are expected for fruitful amalgamation of item **5a**. Blue LED intensity changes were also utilized to determine the ideal settings. The best outcomes, as indicated by the analysts, were obtained when blue LED (12 W) was utilized (Table 2, entry 10). Many substrates were tried under ideal circumstances (Table 3 and Scheme 1). It is vital to take note that the aniline substituent significantly affected the response’s result (Table 3). Both electron-donating and electron-withdrawing functional groups functioned admirably. The yield of all aliphatic and benzylic amines is incredibly high. The reaction patterns of dimethyl acetylenedicarboxylate (DMAD) and diethyl acetylenedicarboxylate (DEAD) were similar.

**TABLE 1:** Photocatalyst optimization table is supplied for **5a** production*^a^*.

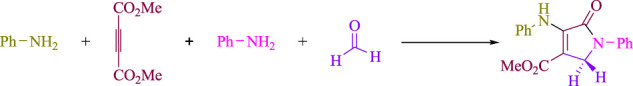				
Entry	Photocatalyst	Solvent (3 ml)	Time (min)	Isolated yield (%)
1	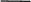	EtOH	60	Trace
2	Acridine yellow G (1.0 mol%)	EtOH	20	78
**3**	**Acridine yellow G (1.5 mol%)**	**EtOH**	**20**	**97**
4	Acridine yellow G (2.0 mol%)	EtOH	20	97
5	Xanthene (1.5 mol%)	EtOH	20	47
6	Riboflavin (1.5 mol%)	EtOH	20	61
7	9*H*-Xanthen-9-one (1.5 mol%)	EtOH	20	49
8	Rhodamine B (1.5 mol%)	EtOH	20	57
9	Alizarin (1.5 mol%)	EtOH	20	42
10	Phenanthrenequinone (1.5 mol%)	EtOH	20	44
11	Acenaphthenequinone (1.5 mol%)	EtOH	20	46
12	Rose bengal (1.5 mol%)	EtOH	20	68
13	Erythrosin B (1.5 mol%)	EtOH	20	65
14	Fluorescein (1.5 mol%)	EtOH	20	67

a Reaction conditions: at rt, formaldehyde (1.5 mmol), aniline (2 mmol), and dimethyl acetylenedicarboxylate (DMAD) (1 mmol) in EtOH, were utilized, alongside a blue LED (12 W) and an assortment of photocatalysts.

The bold parts are the results of the present study.

**TABLE 2:** Table of solvent and visible light optimization is provided for **5a** synthesis*^a^*.

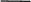 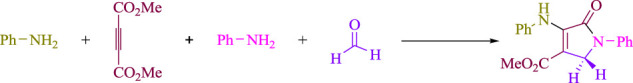				
Entry	Light source	Solvent (3 ml)	Time (min)	Isolated yield (%)
1	—	EtOH	60	Trace
2	Blue light (10 W)	EtOH	20	91
3	Blue light (18 W)	EtOH	20	97
4	White light (12 W)	EtOH	20	84
5	Green light (12 W)	EtOH	20	89
6	Blue light (12 W)	MeOH	20	79
7	Blue light (12 W)	H_2_O	40	42
8	Blue light (12 W)	—	40	45
9	Blue light (12 W)	DMF	55	28
**10**	**Blue light (12 W)**	**EtOH**	**20**	**97**
11	Blue light (12 W)	DMSO	50	31
12	Blue light (12 W)	EtOAc	20	68
13	Blue light (12 W)	THF	55	36
14	Blue light (12 W)	CH_3_CN	20	65
15	Blue light (12 W)	DCM	55	12
16	Blue light (12 W)	toluene	40	38

a Reaction conditions: formaldehyde (1.5 mmol), aniline (2 mmol), and dimethyl acetylenedicarboxylate (DMAD) (1 mmol) were added to AYG (1.5 mol%) at room temperature.

The bold parts are the results of the present study.

**TABLE 3:** Photoexcited AYG produces polyfunctionalized dihydro-2-oxypyrroles.

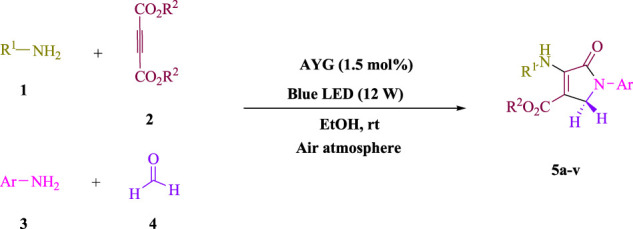	
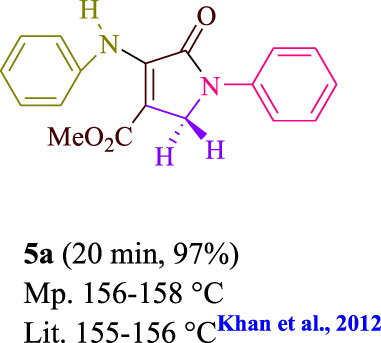	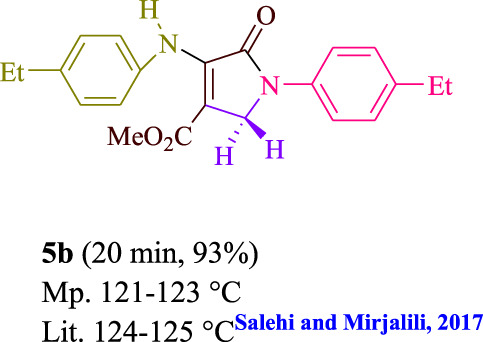
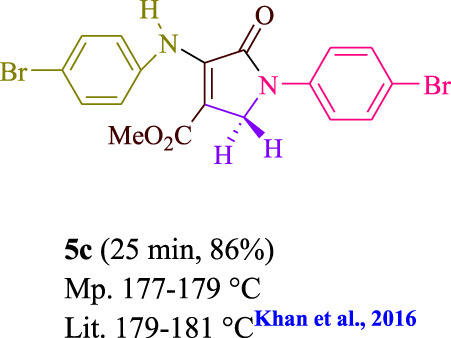	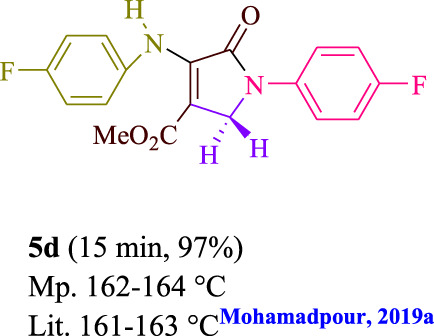
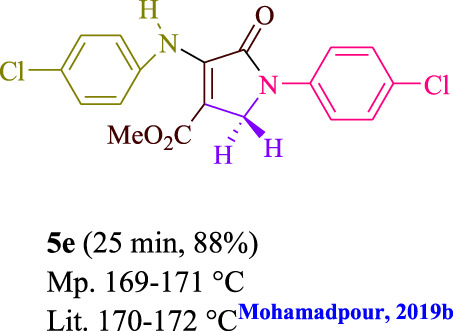	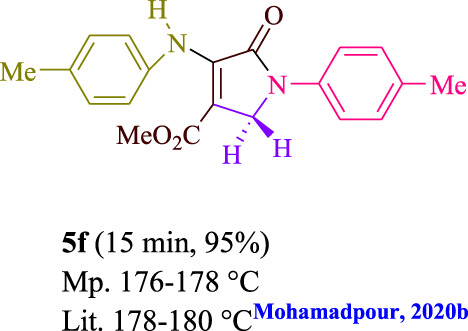
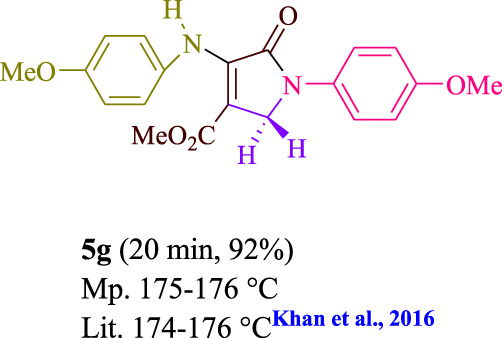 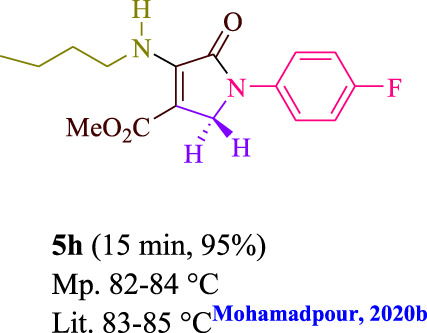	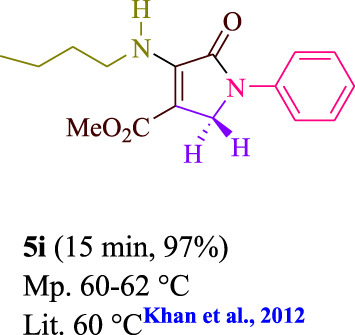 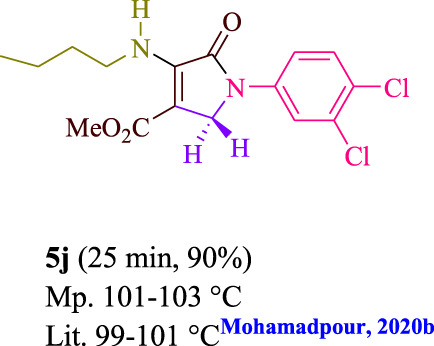
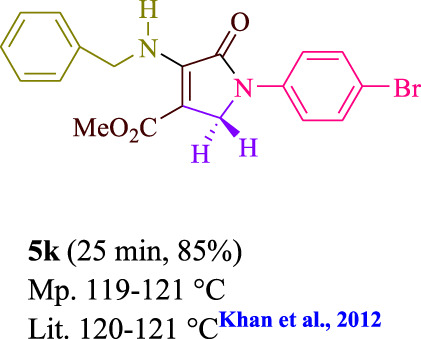 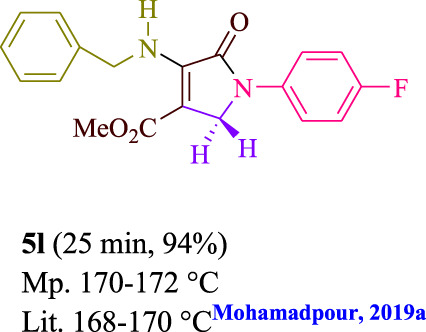	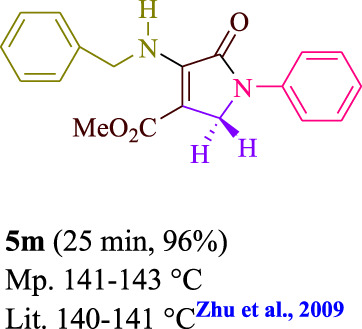 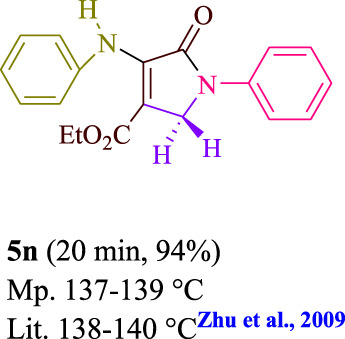
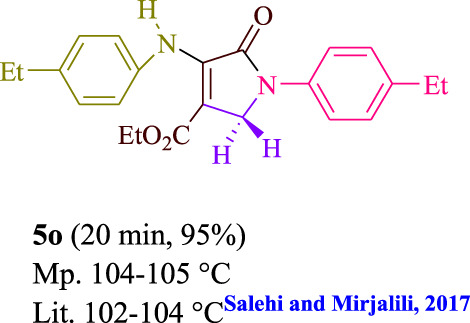 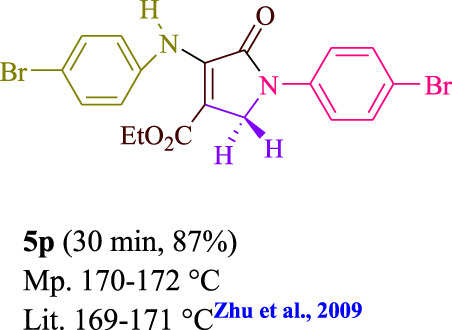	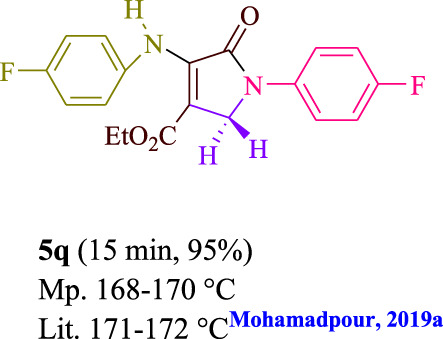 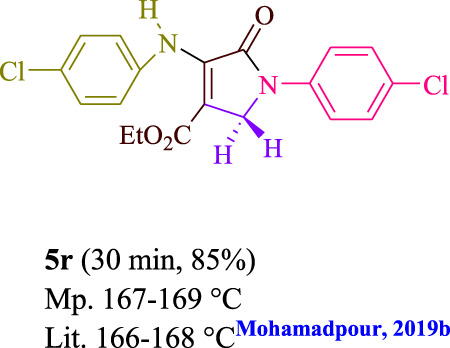
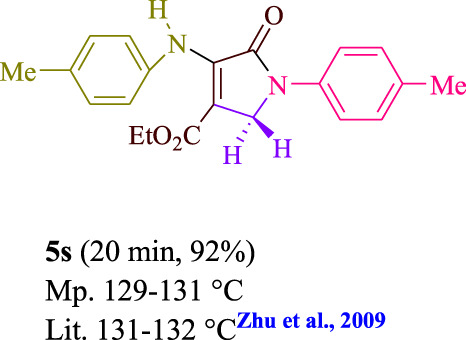	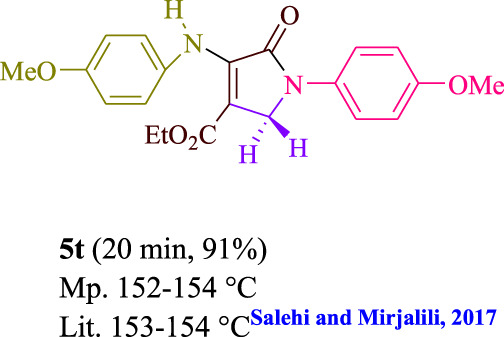
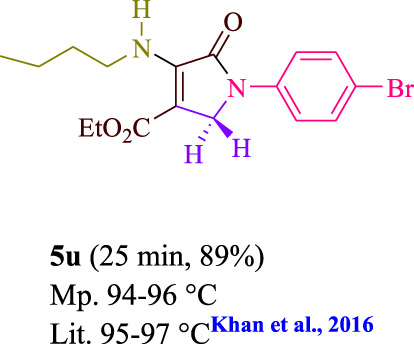	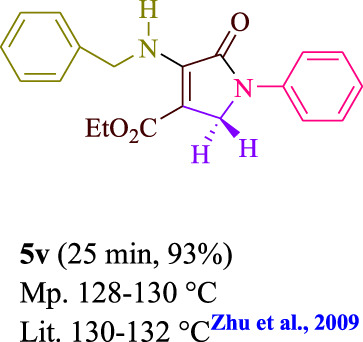

Table 4 additionally remembers information for turnover frequency (TOF) and turnover number (TON). The higher the TON and TOF mathematical qualities, the less the catalyst is utilized and the more prominent the yield gets, and as the worth ascents, the impetus turns out to be more viable.

**TABLE 4:** For polyfunctionalized dihydro-2-oxypyrroles, the determined turnover number (TON) and the turnover frequency (TOF).

Entry	Product	TON	TOF	Entry	Product	TON	TOF
1	**5a**	64.6	3.23	12	**5L**	62.6	2.50
2	**5b**	62	3.1	13	**5m**	64	2.56
3	**5c**	57.3	2.29	14	**5n**	62.6	3.13
4	**5d**	64.6	4.30	15	**5o**	63.3	3.16
5	**5e**	58.6	2.34	16	**5p**	58	1.93
6	**5f**	63.3	4.22	17	**5q**	63.3	4.22
7	**5g**	61.3	3.06	18	**5r**	56.6	1.88
8	**5h**	63.3	4.22	19	**5s**	61.3	3.06
9	**5i**	64.6	4.30	20	**5t**	60.6	3.03
10	**5j**	60	2.4	21	**5u**	59.3	2.37
11	**5k**	56.6	2.26	22	**5v**	62	2.48

The bold parts are the results of the present study.

**SCHEME 2:**
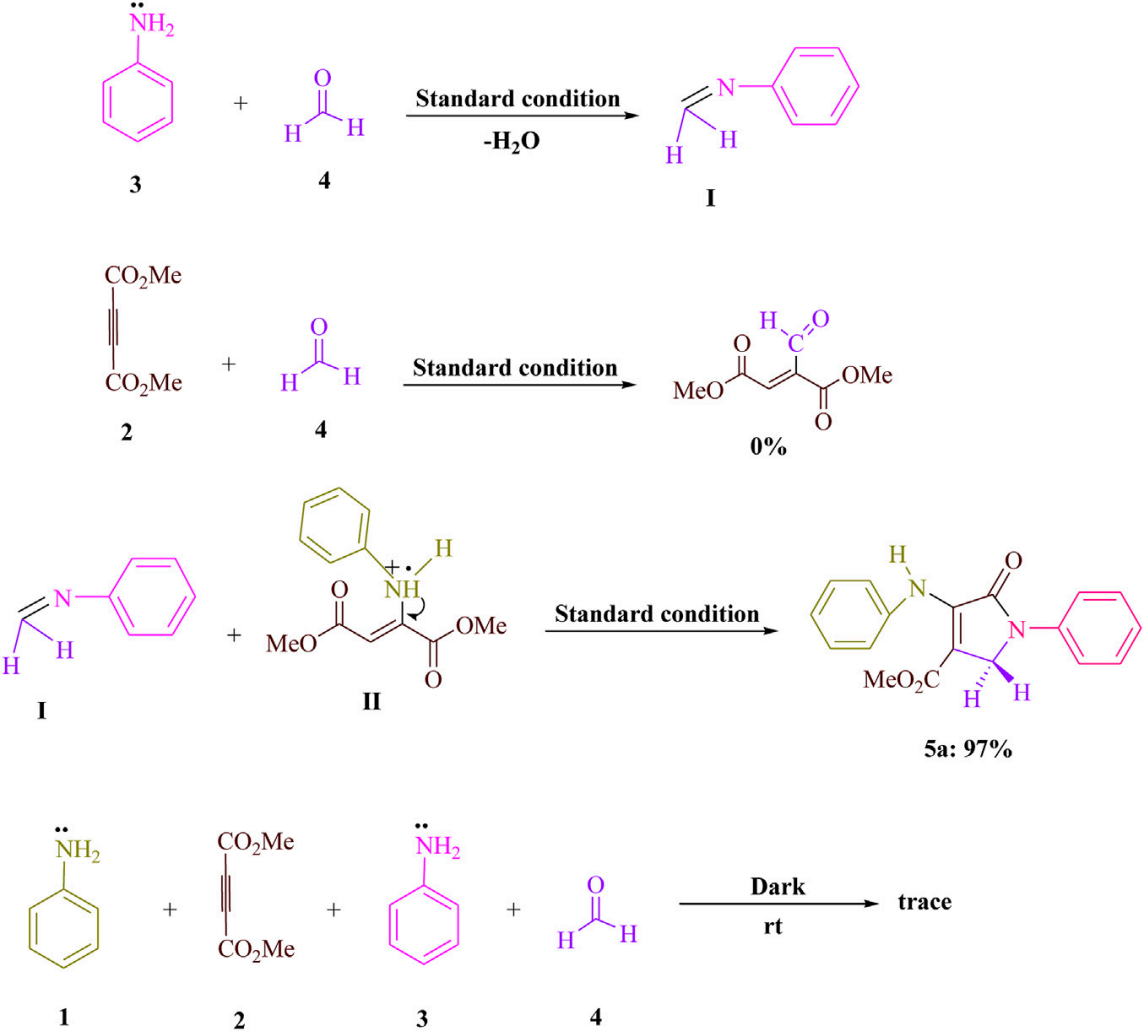
Significant control reads up for grasping the component of formaldehyde (**4**, 1.5 mmol), dimethyl acetylenedicarboxylate (DMAD) (**2**, 1 mmol), and aniline (**1** and **3**, 2 mmol) condensations.

**SCHEME 3:**
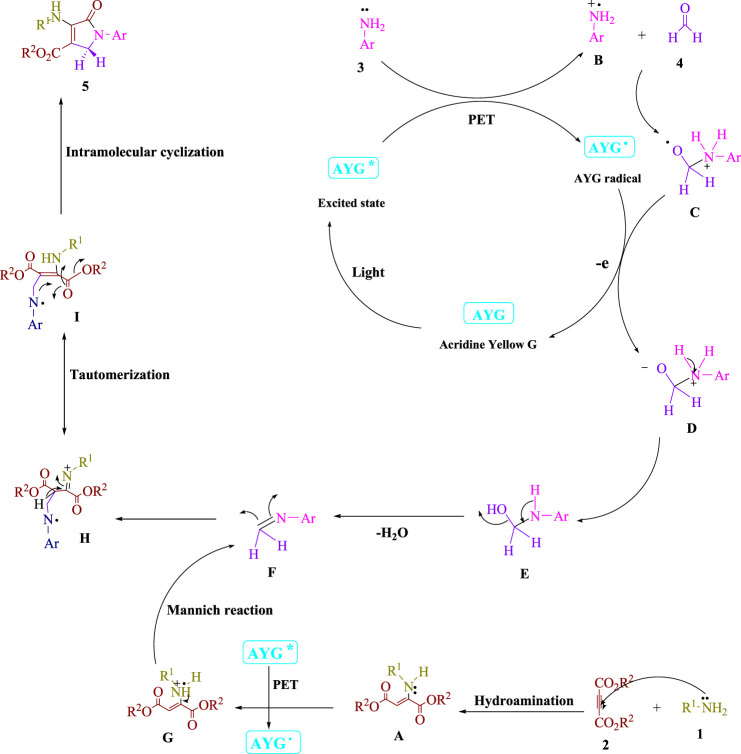
Manufacture of polyfunctionalized dihydro-2-oxypyrroles was described using a mechanistic process.

To acquire knowledge of the response system of this noticeable light-advanced four-component condensation, various control tests were performed. As displayed in Scheme 2, the condensation of aniline **3**) with formaldehyde **4**) was performed under standard circumstances (AYG in EtOH under blue LED) with the end of H_2_O to get the related imine (**I**). When dimethyl acetylenedicarboxylate (DMAD) **2**) was responded with formaldehyde **4**) under indistinguishable response conditions, no item was created. For the condensation of imine (**I**) and enamine radical (**II**), the yield for **5a** was 97%. A trace of the corresponding product **5a** was obtained when the reaction was carried out in the dark. Scheme 3 provides a possible reaction route in the presence of AYG after reviewing the findings of this experiment.

The recommended technique is depicted in Scheme 3. This broadly accessible AYG utilizes noticeable light as a wellspring of sustainable power to develop reactant frameworks that use the PET pathway. This process can be accelerated with visible light energy. Enamine (**A**) is produced through the Michael reaction between amine **1**) and dialkyl acetylenedicarboxylate (**2**). To boost the visible-light-induced AYG^*^, the aniline radical (**B**) is produced utilizing a PET method and visible light irradiation. After that, the radical cation (**B**) reacts with formaldehyde **4**) to create a radical cation (**C**). The electron transfer (ET) process between the radical adduct (**C**) and the AYG radical produces the intermediate (**D**) and ground-state AYG. Then, from (**E**), an H_2_O molecule is removed, leaving intermediate (**F**). To boost the visible-light-induced AYG^*^, the enamine radical (**G**) is produced utilizing a PET approach. A Mannich reaction occurs between an activated imine (**F**) and an enamine radical (**G**), resulting in an intermediate (**H**) that changes into a more stable tautomeric form (**I**). In the final phase, the intramolecular cyclization in intermediate (**I**) tautomerizes into polyfunctionalized dihydro-2-oxypyrroles (**5**).

## Conclusion

A radical synthesis of polyfunctionalized dihydro-2-oxypyrroles utilizing AYG dye as a photo-induced electron transfer photocatalyst was studied. Visible light is used as a renewable energy source in an ethanol solution at room temperature in an air environment. The utilization of a minimal amount of photocatalyst, brilliant yields, a response with high proficiency, stable response conditions, and a renewable energy source, and a fast methodology without the utilization of harmful solvents or impetuses are the clearest benefits of this green convention. Chromatographic purging was not needed. As per a multigram scale response of model substrates, this response can be increased without compromising the result. Therefore, this innovation offers colossal advantages regarding both gathering modern requirements and settling ecological worries.

## Data Availability

The datasets presented in this study can be found in online repositories. The names of the repository/repositories and accession number(s) can be found in the article/Supplementary Material.
