# Supplementary material for: Acridine yellow G (AYG) as a photo-induced electron transfer (PET) photocatalyst employed for the radical Michael–Mannich cyclocondensation of imines
Source: Front Chem. 2022 Oct 6;10:1015330. doi: 10.3389/fchem.2022.1015330 (PMC9590109; doi:10.3389/fchem.2022.1015330)
Supplement: Supplementary file 1 [file DataSheet1.PDF]

Supporting information

Acridine yellow G (AYG) as a photo-induced electron transfer (PET) photocatalyst employed for the radical Michael-Mannich cyclocondensation of imines

Farzaneh Mohamadpour [*]

School of Engineering, Apadana Institute of Higher Education, Shiraz, Iran

* Corresponding author. mohamadpour.f.7@gmail.com

Methyl 4-(4-bromophenylamino)-1-(4-bromophenyl)-2,5-dihydro-5-oxo-1H-pyrrole-3-carboxylate (5c)

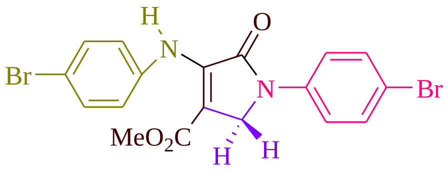

Yield: 86%; M.p. 177-179 °C; ^{1}HNMR (300 MHz, $CDCl_3$): 3.81 (3H, s, OCH_3), 4.52 (2H, s, $\underline{CH_2}$–N), 7.04 (2H, d, *J*= 11.2 Hz, ArH), 7.46 (2H, d, *J*= 11.6 Hz, ArH), 7.53 (2H, d, *J*= 12.0 Hz, ArH), 7.71 (2H, d, *J*= 12.0 Hz, ArH), 8.06 (1H, s, NH) ppm.

Methyl 3-(butylamino)-2,5-dihydro-2-oxo-1-phenyl-1Hpyrrole-4-carboxylate (5i)

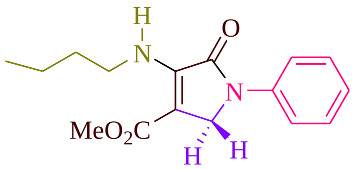

Yield: 97%; M.p. 60-62°C; ^{1}HNMR (300 MHz, $CDCl_3$): 0.98 (3H, t, *J*= 9.6 Hz, CH_3), 1.42 (2H, sextet, *J*= 9.2 Hz, CH_2), 1.64 (2H, quintet, *J*= 9.0 Hz, CH_2), 3.82 (3H, s, OCH_3), 3.89 (2H, t, *J*= 9.2 Hz, $\underline{CH_2}$–NH), 4.44 (2H, s, $\underline{CH_2}$–N), 6.67 (1H, br s, NH), 7.19-7.24 (1H, m, ArH), 7.40-7.45 (2H, m, ArH), 7.79 (2H, d, *J*= 10.4 Hz, ArH) ppm.

Ethyl 1-phenyl-3-(phenylamino)-2,5-dihydro-2-oxo-1Hpyrrole-4-carboxylate (5n)

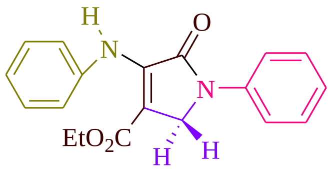

Yield: 94%; M.p. 137-139 °C; ^{1}HNMR (300 MHz, $CDCl_3$): 1.23 (3H, t, *J*= 9.6 Hz, $OCH_2\underline{CH_3}$), 4.24 (2H, q, *J*= 9.6 Hz, $O\underline{CH_2}CH_3$), 4.58 (2H, s, $\underline{CH_2}$–N), 7.16–7.25 (4H, m, ArH), 7.29-7.37 (2H, m, ArH), 7.40-7.46 (2H, m, ArH), 7.84 (2H, d, *J*= 11.6 Hz, ArH), 8.01 (1H, s, NH) ppm.
